# Exploring Medical Students’ Learning Around Uncertainty Management Using a Digital Educational Escape Room: A Design-based Research Approach

**DOI:** 10.5334/pme.844

**Published:** 2023-03-20

**Authors:** Jenny Moffett, Dara Cassidy, Naoise Collins, Jan Illing, Marco Antonio de Carvalho Filho, Harold Bok

**Affiliations:** 1 HPEC Health Professions’ Education Centre, RCSI University of Medicine and Health Sciences, 123 St. Stephen’s Green, Dublin, Ireland; 2 Department of Visual and Human-Centred Computing, Dundalk Institute of Technology, Dublin Rd, Marshes Upper, Dundalk, Ireland; 3 Wenckebach Institute, Health Profession Education Research, Faculty of Medical Sciences, University Medical Center Groningen, Groningen, The Netherlands; 4 Department of Population Health Sciences, Faculty of Veterinary Medicine, Utrecht University, Utrecht, The Netherlands

## Abstract

**Introduction:**

Medical professionals meet many transitions during their careers, and must learn to adjust rapidly to unfamiliar workplaces and teams. This study investigated the use of a digital educational escape room (DEER) in facilitating medical students’ learning around managing uncertainty in transitioning from classroom to clinical placement.

**Methods:**

We used design-based research to explore the design, build, and test of a DEER, as well as gain insight into how these novel learning environments work, using Community of Inquiry (CoI) as a guiding conceptual framework. This study represented a mixed methods pilot test of a prototype DEER. Twenty-two medical students agreed to participate, and data were collected through qualitative (i.e., focus groups, game-play observations) and quantitative (i.e., questionnaires) methods.

**Results:**

Eighty-two per cent of participants agreed or strongly agreed that the DEER supported their learning around uncertainty. Participants offered diverse examples of how the game had facilitated new insights on, and approaches to, uncertainty. With respect to the learning environment, multiple indicators and examples of the three domains of CoI – cognitive, teaching and social presence – were observed.

**Discussion:**

Our findings suggested that DEERs offer a valuable online learning environment for students to engage with complex and emotion-provoking challenges, such as those experienced at transitions. The study also suggested that CoI can be applied to the design, implementation, and evaluation of DEER learning environments, and we have proposed a set of design principles that may offer guidance here.

## Introduction

Medical professionals meet many transitions during their careers, and must learn to adjust rapidly to unfamiliar workplaces and new teams. Such profound changes begin in medical school; an early and important example of this is the transition from pre-clinical to clinical training. This step into ‘real-world’ medicine represents an exciting and rewarding time for medical students [1]. However, it is also a step into the unknown, with the potential to evoke experiences of stress and uncertainty [2,3]. Although many supports exist which address the knowledge and practical skills needed for clinical placements (e.g., special-purpose courses, clinical skills training), these can fall short in preparing students for ‘the dynamics of a new environment, which itself is unstable’ [3, p.566]. With healthcare practice becoming increasingly complex and unpredictable [4], it is important to better prepare students to engage with dynamic clinical learning environments.

In recent years, there has been an increased interest in how medical professionals manage uncertainty, both at transitions and more generally [5]. The evidence highlights that health professionals’ responses to uncertainty can influence their decision-making skills [6], attitudes to patients [7], career choices [8], and experiences of work-related stress [9,10]. More recent research also suggests that it may be possible to train medical students to prepare for uncertainty [11]. Clinical debriefs, simulations, and peer-to-peer conversations have been proposed as pedagogical approaches that may help students to better manage the uncertainty of clinical practice [12,13]; however, there is little empirical research in this domain.

This study explores the use of a type of simulation-based educational game known as an escape room to facilitate medical students’ learning around uncertainty experienced at the transition from classroom into clinical settings. Escape rooms are ‘live-action team-based game where players discover clues, solve puzzles, and accomplish tasks in one or more rooms in order to accomplish a specific goal… in a limited amount of time’ [14]. Educational escape rooms have rapidly become popular within health professions’ education [15]. A variety of studies have explored the capacity of escape rooms to facilitate learning in clinical [16,17,18,19,20] and professional skills [21,22,23] domains. Research, however, is at an early stage with relatively little known about how learning takes place within these novel environments [24].

Escape rooms can be held within face-to-face or virtual learning environments where, in the latter case, they are referred to as digital educational escape rooms (DEERs). In this study, we built a DEER in order to explore how this learning environment might be used to facilitate medical students’ learning around uncertainty, as well as to gain more general insight as to how escape rooms work. We selected the Community of Inquiry (CoI) model [25] as a guiding conceptual framework, and a lens with which to investigate the DEER learning environment. CoI is a widely studied online learning model [26,27], that can help researchers to conceptualise ‘the educational transaction and processes of learning’ in online settings [28, p.9]. The framework (Table 1) proposes that meaningful online learning arises through the development of three overlapping domains [29]:

- Cognitive presence (i.e., the extent to which learners are able to construct meaning through sustained reflection and discourse [25]);
- Teaching presence (i.e., the ‘design, facilitation, and direction of cognitive and social processes for the purpose of realizing personally meaningful and educational worthwhile learning outcomes’ [30, p.1]); and,
- Social presence (i.e., ‘the ability of learners to project themselves socially and affectively into a community of inquiry’ [31, p.4]).

**Table 1:** Community of inquiry elements, categories and indicators (adapted from Garrison & Arbaugh, 2007. [32]).


ELEMENTS	CATEGORIES	INDICATORS

**Cognitive**	Triggering event	Having a sense of puzzlement

	Exploration	Exchanging information

	Integration	Connecting ideas

	Resolution	Applying new ideas

**Teaching**	Design and organisation	Setting curriculum and methods

	Facilitation of discourse	Sharing personal meaning

	Direct instruction	Focusing discussion

**Social**	Open communication	Enabling risk-free communication

	Group cohesion	Encouraging collaboration

	Affective expression	Expressing emotions, camaraderie


CoI adopts a collaborative-constructivist stance [33], making it a framework of particular interest for the team-based DEER learning environment [34,35]. However, there is limited empirical research here too. Thus, our research questions for this study were:

- What are medical students’ perspectives on the use of a digital educational escape room to facilitate learning around managing uncertainty at the transition from classroom to clinical placement?
- What impact, if any, does a DEER have on medical students’ uncertainty tolerance?
- Does CoI facilitate our understanding of DEER learning environments, and, if so, what indicators of social presence, teaching presence and cognitive presence exist?

To explore these research questions, we used a design-based research (DBR) approach. DBR is ‘a systematic but flexible methodology aimed to improve educational practices through iterative analysis, design, development, and implementation, based on collaboration among researchers and practitioners in real-world settings’ [36]. A key tenet of DBR is that it holds dual goals: the research should facilitate the development of a specific innovation or intervention, whilst also testing and refining theories to gain insight into complex learning environments [37]. Although there is great variety in how DBR is implemented, this approach typically involves four stages: analysis of the problem, design of solutions, testing and iteration, and reflection [38]. In this study, we used DBR to design, build and test our DEER in an online setting whilst simultaneously furthering our understanding of the applications of CoI in this context.

## Methods

### Study design

**Figure 1:**
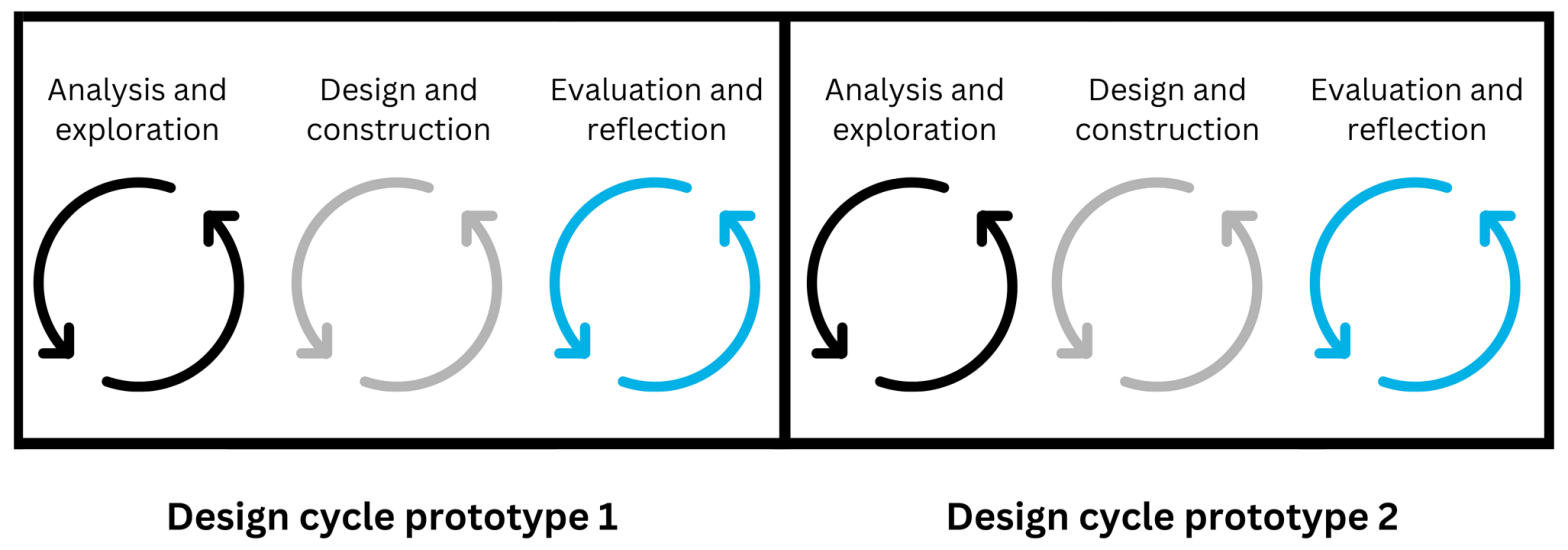
Data from a preliminary design cycle was used to inform the build for a second prototype (adapted from McKenney & Reeves, 2012. [42]).

DBR involves the development and evaluation of multiple prototypes. An initial prototype DEER underwent evaluation [39] and data from that design cycle was used to inform the build for this second prototype (Figure 1). The current study explores a design cycle where the second prototype escape room was pilot-tested using a convergent parallel mixed methods study design [40]. We used qualitative (i.e., focus groups, game-play observations) and quantitative (i.e., questionnaires) data collection methods, with an emphasis on the qualitative strand [41]. Ethical approval for the study was granted by the RCSI Research and Ethics Committee, RCSI University of Medicine and Health Sciences (ID 202103004).

### Context

The study took place at RCSI University of Medicine and Health Sciences, a culturally diverse institution with over 4,000 students from 90 different countries. The university offers a direct-entry medical programme with two pre-clinical (Years 1–2) and three clinical years (Years 3–5). Our study population consisted of students enrolled in Year 2 of the programme in advance of their commencing clinical placements. All students within this cohort were eligible to participate, and recruitment was promoted via university email and social media. An incentive to take part, entry into a draw for a book voucher, was offered.

Study participants were invited to play a prototype DEER in October 2021. This prototype had been build using draft principles derived from the first design cycle and a review of the CoI research literature (Table 2). The DEER was designed to be played by small groups (4–5) of students, and it was intended that students would work together to overcome ambiguity, solve puzzles and ‘escape’ a fictional creepy hospital [39]. The DEER consisted of ten puzzles, including numerical, word-based, logic, and general knowledge formats, and three in-game reflections, which were built on an interactive content authoring platform (Genially; Madrid, Spain). Individual puzzles were designed to align with sources of uncertainty in healthcare that have been identified by Han *et al*. [43]. This meant that participants met puzzles which involved managing complex information, recognising ambiguity, and working with the different outcomes that can emerge in medicine (i.e., patient gets better, or patient does not). Although participants could follow different pathways within the DEER, all groups needed to complete a final, culminating ‘meta-puzzle’ to complete the game.

**Table 2:** Design principles for DEERs that are underpinned by Community of Inquiry (CoI).


COI PRESENCE	DESIGN PRINCIPLES	REFERENCES

Cognitive	Use an engaging storyline that evokes curiosity for learners Align escape room puzzles with educational learning outcomes Provide challenging puzzles that provoke shared reflection	Garrison, 2016 [44]; Garrison, 1999 [45]; Redmond, 2014 [46]

Teaching	Provide clear instructions to learners before game Use facilitation skills to establish a safe, supportive learning environment Offer scaffolded support to learners throughout (e.g., pre-brief, hint strategy, technical support, de-brief)	Cheng, 2020 [47]; Garrison, 2016 [44]; McKerlich, 2007 [48]; Shea, 2010 [49]

Social	Use web-conferencing software with breakout room capability to facilitate small group interactions Employ collaborative rather than competitive game strategies (e.g., escape against clock rather than ‘first team to escape wins’) Use puzzles to evoke emotions such as confusion and excitement	Fayram, 2017 [50]; Garrison, 2016 [44]; Lowenthal, 2014 [51]; Moallem, 2015 [52]


**Figure 2:**
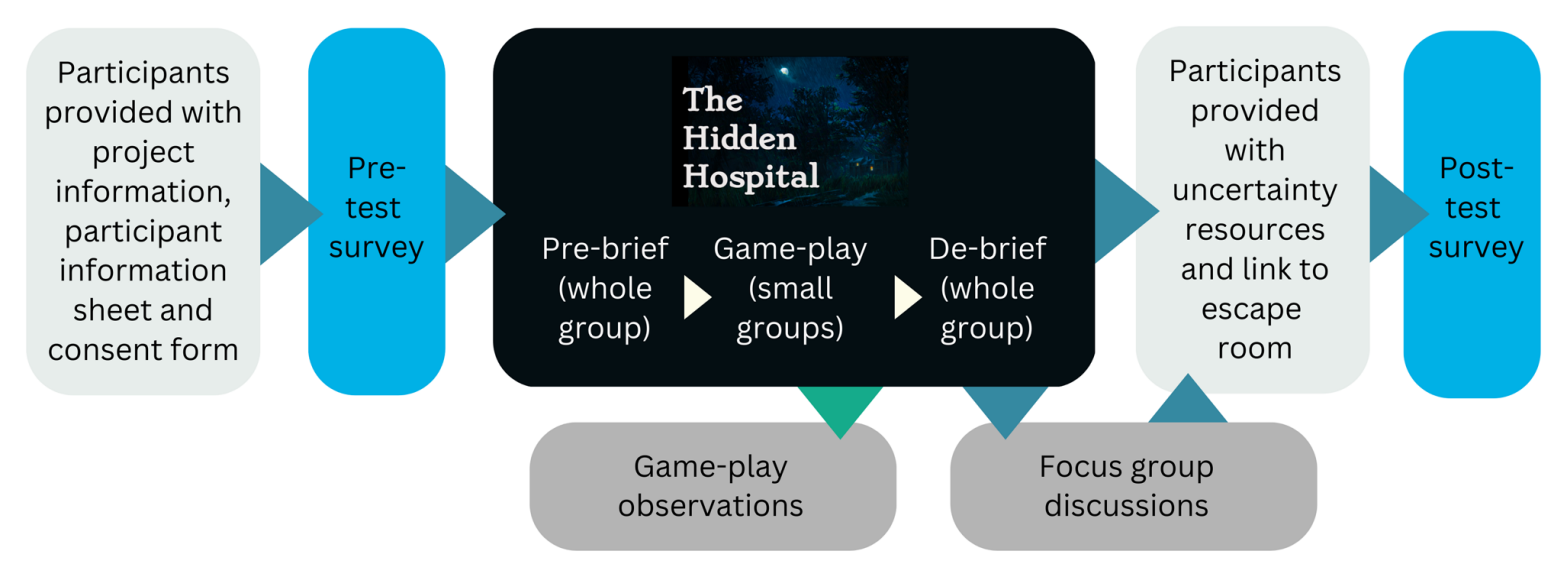
Flow chart of the study design.

Prior to game-play, participants were given details of the DEER, a participant information sheet and a consent form (Figure 2). On the day of game-play, participants joined the session via Microsoft Teams (Redmond, WA, USA). There was a short introduction, or pre-brief, before participants were asked to join breakout rooms and begin the activity. The pre-brief aimed to establish psychological safety by providing clear instructions for game play, as well emphasising the fun element and the availability of help for overcoming “roadblocks” encountered during the game [47]. Each group was allocated 50 minutes of game play, and participants were directed to play as a team, appointing leaders to ‘share screens’ and input answers. After the allocated time, breakout rooms were closed, and a de-brief with the full cohort of students was held. The de-brief was designed to allow participants an opportunity to disclose and discuss the uncertainties that arose for them, as well as other experiences that they felt were important. The de-brief also offered a space for the participants to engage in shared reflection around the key learning outcomes from game play, including the in-game reflections. Finally, an email with uncertainty management resources and a link to the DEER was sent to participants after the session.

### Data collection

#### Qualitative data collection

Qualitative data were collected during game-play and immediately afterwards through focus group discussions. Game-play and break-out rooms were video-recorded and the audio component transcribed. Text from the session web-chat as well as observational data (e.g., the actions of the participants) were also recorded. The focus group discussions, facilitated by experienced researchers using a pre-determined question guide (Appendix A), were also video-recorded and the audio transcribed. Focus group participants were also invited to submit text responses via a digital noticeboard, Padlet, to ensure everyone had the opportunity to provide feedback (Padlet; San Francisco, CA, USA), which were collected.

#### Quantitative data collection

Quantitative data were collected before and after the game-play session through use of pre- and post-intervention questionnaires, via an online survey platform (SurveyMonkey; San Mateo, CA, USA). Data collection was intended to capture any impact of game-play on participants’ uncertainty tolerance. The pre-intervention questionnaire (Appendix B) consisted of: the Intolerance of Uncertainty Scale (Short Form) (IUS-12), a 12-item questionnaire which assesses individuals’ perceptions of uncertainty and which has previously demonstrated high internal consistency (α = 0.91) with medical student cohorts [53]; the Tolerance for Ambiguity (TFA) Scale, a 7-item questionnaire which assesses individuals’ tolerance of general uncertainty in life and which has demonstrated acceptable internal consistency with cohorts of medical students (α = 0.75) [54]; and, a set of demographic questions. The post-intervention questionnaire (Appendix C) consisted of repeats of the IUS-12 and TFA, alongside a 12-item escape room perception survey adapted from Eukel *et al*. [55].

### Data analysis

#### Qualitative data analysis

Two separate qualitative data analyses were carried out. The first analysis explored the focus group transcriptions and digital noticeboard text. Here, data were combined and organised using NVivo 12 (QSR International; Melbourne, Australia), and examined using a reflexive thematic analysis approach [56]. The researchers used an initial inductive step to understand the experiences of the students in relation to the escape room. JM listened to the audio data, and then read and re-read the transcribed recordings. JM then created initial codes, which were specifically related to participants’ perspectives of using a DEER to facilitate learning around uncertainty. JM then applied a subsequent step of deductive analysis whereby the data was examined with respect to the social, cognitive and teaching presences of CoI. Following several passes through the data, themes were identified, refined and re-organised before final agreement with the research team (JM, DC & JI).

The second analysis explored the game-play transcriptions, web-chat and qualitative observational data. Here, JM and DC used a CoI instrument adapted from McKerlich & Anderson [48] to examine the data. This involved viewing the session videos twice, reading and re-reading the session transcripts and web-chat text, before discussing and documenting indicators and examples of social, cognitive and teaching presences. The researchers drew on existing CoI research [49,51,57] to help define boundaries around the presences.

#### Quantitative data analysis

Quantitative data were analysed in two stages. First, the pre- and post-intervention surveys items were analysed. Internal consistency was assessed by calculating Cronbach’s coefficient alpha for each [58], and a Shapiro-Wilks test was used to assess the normality of the resulting data. A paired-design t-test was used to determine if there was a significant difference between the scores on the IUS-12 scale and the TFA scale pre- versus post-intervention. A separate test was carried out for each of the scales and alpha was set at 0.05.

Second, a one-sample t-test and descriptive statistics were used to explore responses to the escape room perception survey. The perception survey was measured on a five-point Likert scale ranging from ‘1 = strongly disagree’ to ‘5 = strongly agree’, with items 9 and 10 of the survey reverse-scored. The one-sample t-test assessed whether students’ mean (SD) perception score was significantly different to the mean value of the scale, ‘3 = not agree nor disagree’. All statistical analyses were carried out using STATA statistical package version 17 (StataCorp; Texas, USA).

### Reflection

As a final stage of data analysis, the research team (HB, MDF, JM and DC) met to discuss the data in relation to the initial DEER design principles. The researchers examined the data through the lens of the CoI framework and engaged in shared reflection, with the aim of co-constructing an updated set of design principles.

## Results

Our results are organised in two sections. First, we report findings that relate to our first and second research questions, i.e. exploring the use of a DEER in relation to medical students’ learning around, and tolerance of, uncertainty. Second, we report findings that relate to our third research question (i.e., investigating the CoI as a framework of relevance in understanding DEER learning environments). Twenty-two second year pre-clinical undergraduate medical students (10 female and 12 male students) agreed to participate in the study. Participant quotes, with details on focus group, gender and participant number (e.g., FG1F1), have been provided.

### Using a DEER in relation to medical students’ learning around, and tolerance of, uncertainty

#### Qualitative data

Ten participants (4 female; 6 male students) participated in two focus group discussions. Data analysis of the focus groups identified two themes that related to the participants’ perspectives on using a DEER to facilitate learning around uncertainty: affective experiences of uncertainty, and building approaches to uncertainty.

The participants highlighted that the DEER learning environment provided multiple opportunities for affective experiences of uncertainty. They noted that playing the game felt inherently uncertain due to the challenges of the puzzles and the ambiguous clues. Others felt unsure about what the game would entail, and whether it would represent a good use of their time. Further to this, participants reported uncertainty in relation to working with new and unfamiliar colleagues. Some participants expressed self-doubt and a sense of vulnerability in relation to their abilities (i.e., whether or not they would be able to complete the game, or contribute to the team). ‘*I don’t know if I need tonnes of outside knowledge and all? I don’t want to be the weak person, throwing out stuff that’s completely left field and not at all correct.*’ (FG1F1)

One group reported experiences of uncertainty due to a technology breakdown (i.e., lagging internet connection). Overall, participants spoke of uncertainty in terms of a variety of different emotional states including anxiety, frustration, curiosity and excitement. ‘*I’ve never actually ever come across something like this escape room… I was pretty curious and anxious, like what it is we will actually do*?’ (FG1M1)

The participants discussed ways in which the DEER had helped them to think differently about uncertainty. They highlighted new strategies in managing uncertainty, such as adopting a team approach (i.e., harnessing different perspectives). The validation and support of others helped them to propose ideas and solutions, despite feeling unsure. ‘*A lot of moments I was confused and didn’t know what to do and they backed me up. Individually, we didn’t know everything. This is something we all need to learn, it’s an important student experience. It was like a metaphor for diagnosing patients.*’ (FG1M2)

Participants also reported that the game had helped them to engage with critical thinking and creative approaches to problem solving. Others alluded to shifts from negative to more positive mind-sets around uncertainty. ‘*There will be times when we will be uncertain so it shouldn’t be a factor that makes us feel uncomfortable. It should be a motivating factor to learn more.*’ (FG1M3)

However, not all participants agreed that they had learned about uncertainty. Some felt that the puzzles did not reflect the uncertainty experienced in real-world, clinical practice. Others commented that the learning was not linked to their course work, and thus seemed less relevant to them. These views were predominant within the group who had experienced technology problems.

‘*I just feel like, have we really learnt anything by playing the game?*’ (FG2F1)

#### Quantitative data

Sixteen participants (16/22, 73% of the study cohort) completed both the pre-intervention and post-intervention questionnaires. The reliability was high for the IUS-12 scale (Cronbach’s alpha = 0.89) and acceptable for the TFA-scale (Cronbach’s alpha = 0.74). The data were found to be normally distributed on the Shapiro-Wilks test. No significant difference in Intolerance of Uncertainty (t = 0, df = 15, p-value = 1) nor Tolerance of Ambiguity (t = –0.81, df = 15, p-value = 0.43) was detected between the pre-intervention and post-intervention groups.

With respect to the escape room perceptions survey, 17 participants submitted responses (77% of the study cohort) (Table 3). The mean perception value for the cohort (m = 3.99 +/– 0.59 sd) on a five-point evaluation scale was significantly higher than the neutral point (3) of the evaluation scale (t = 6.98, df = 16, p < 0.01). This suggests that the students’ perceived learning through the escape room was strongly positive.

**Table 3:** Escape room perception survey (n = 17) (adapted from Eukel *et al*. [55]).


ITEM	MEAN (SD)	STRONGLY DISAGREE (%)	DISAGREE (%)	NEUTRAL (%)	AGREE (%)	STRONGLY AGREE (%)

1. The escape room encouraged me to think about material in a new way	3.7 (0.3)	5.9	5.9	23.5	41.2	23.5

2. I would recommend this activity to other students	4.5 (0.2)	0	5.9	0	35.3	58.8

3. I learned from my peers during the uncertainty escape room	4.3 (0.1)	0	0	5.9	58.8	35.3

4. The escape room was an effective way to review the topic of uncertainty	3.6 (0.3)	11.8	11.8	0	58.8	17.6

5. The escape room was an effective way to learn new information related to uncertainty	3.5 (0.3)	5.9	17.6	11.8	52.9	11.8

6. I learn better in a game format than in a lecture	4.6 (0.1)	0	0	0	41.2	58.8

7. The escape room was an effective way to assist my learning around managing uncertainty	3.7 (0.3)	5.9	11.8	5.9	58.8	17.6

8. I feel I was able to engage with my teammates to learn new material	4.0 (0.2)	0	5.9	5.9	70.6	17.6

9. It was difficult for me to focus on learning because I was feeling stressed or overwhelmed	3.9 (0.2)*	29.4	41.2	17.6	11.8	0

10. The non-educational portions (e.g., puzzles, etc.) distracted me from learning about uncertainty	3.4 (0.3)*	11.8	47.0	17.6	11.8	11.8

11. I prefer assembling information from a variety of sources when learning new material	4.1 (0.2)	0	5.9	11.8	47.0	35.3

12. In general, I enjoy playing games (video games, board games, social media games, etc.)	4.8 (0.1)	0	0	0	23.5	76.5


* n.b. Items 9 and 10 were negatively worded and have been reversed-scored during analysis.

The majority of participants (n = 14, 82%) agreed or strongly agreed that the escape room was an effective way to assist their learning around managing uncertainty. Ninety-four per cent of the participants agreed or strongly agreed that they had learned from their peers during the game-play session. Finally, 94% of participants agreed or strongly agreed that they would recommend the game to other students.

### CoI as a framework of relevance in understanding DEER learning environments

Data collected during the focus group discussions and the game-play sessions were categorised according to the presences of CoI: cognitive, teaching and social [25]. These data are presented in Appendix D.

#### Focus group data

Within the focus groups, participants highlighted several aspects of the escape room experience that appeared to be consistent with CoI. With respect to social presence, they reported that the game provided a warm environment that supported team interaction. They felt validated, supported and motivated by each other during game play, and reported a wide range of affective experiences including: curiosity, enjoyment, excitement, fun, pride, relief, satisfaction, annoyance, anxiety, confusion, exasperation, and frustration (Table 3). With respect to teaching presence, participants noted the role of the instructor in: setting the tone for the game; establishing team collaboration; offering clear instructions; providing guidance and technical help; supporting insights around uncertainty; and re-emphasising the game’s learning outcomes (Table 3). One aspect of the game’s design that evoked mixed opinions was the ‘race against the clock’ time strategy. Some participants reported that the time pressure added to the fun, and helped them to establish trust within their team quickly. Others said that time pressure caused them to rush through the game, sometimes progressing without fully understanding a topic. With respect to cognitive presence, there were relatively fewer comments. Although many participants reported that the game had involved them in cognitive effort, there appeared to be variation in how deeply they engaged with the puzzles. Many participants commented that the in-game reflective activities broke their sense of flow and immersivity within the game.

#### Game-play data

Qualitative data collected during game-play also highlighted multiple indicators and examples of cognitive, teaching and social presence within the DEER (Table 3). With respect to cognitive presence, participants seemed to share information, connect ideas and test theories with each other. Cognitive presence appeared to be most salient during puzzle-solving interactions. Teaching presence was observed in the planning and organisation of the DEER as well as through facilitation of discourse and direct instruction, which could be subdivided into facilitator and peer categories. Teaching presence related to the facilitator was dominant in the pre- and de-brief sections, whereas teaching presence related to the participants was dominant within the breakout rooms. Social presence was observed during all stages of the session with multiple examples of open communication, group cohesion and affective expression. With regards to the latter, many overt expressions of uncertainty were observed within the peer interactions.

#### Reflection

Following analysis of the data and a process of shared reflection, the research team co-constructed a list of revised design principles for DEERs that are underpinned by the CoI framework (Table 4).

**Table 4:** Revised design principles for DEERs that are underpinned by Community of Inquiry.


COI PRESENCE	DESIGN PRINCIPLES

Cognitive	Use an engaging storyline that evokes curiosity for learners

	Explicitly align escape room puzzles with meaningful/purposeful learning outcomes

	Provide challenging puzzles aligned with learners’ developmental levels which provoke shared reflection

Teaching	Open the game with a pre-brief which provides clear instructions, encourages engagement and establishes a safe, supportive and playful learning environment

	During the game, maintain learner engagement through responsive facilitation (e.g., technical support), and effective game design (e.g., hint strategy)

	After the game, use a debrief to help learners to make sense of the activity, facilitating the resolution phase of cognitive presence as well as emotional closure for learners

	Encourage engagement and peer learning through consideration of small group size and composition, and team-work strategy

	Assist learners who are not familiar with each other to build rapport (e.g., through introductions and ice-breakers)

	Ensure that game play and the ‘rules of engagement’ align with the intended cognitive process, learners’ behaviour, and learning outcomes

Social	Use web-conferencing software with breakout room capability to facilitate small group interactions

	Employ complementary game strategies, from social collaboration to healthy competition, optimising learners’ engagement

	Use puzzles to evoke emotions that increase arousal and positively impact on cognitive presence


## Discussion

This study sought to explore medical students’ perspectives on the use of a DEER to facilitate learning around managing uncertainty at the transitions from classroom to clinical placement, and what impact, if any, a DEER has on students’ uncertainty tolerance. Our findings suggest that DEERs generate an engaging online learning environment that allows medical students to meet with uncertainty in a safe and constructive manner. Many of these uncertainties appear to resonate with those experienced by medical students at clinical transitions (i.e., making sense of ambiguous information, engaging in decision-making under time pressure, and building trust quickly with unfamiliar people). Although at least some of the uncertainty was evoked through the novelty of the DEER, which may decrease as students become more acquainted with such strategies, the game seemed to provoke relevant affective states and offer a supportive environment that facilitated shared disclosure.

Our findings also suggest that the DEER had facilitated learning around uncertainty management. The majority of students perceived that the DEER had assisted their learning, whilst the focus group discussions revealed examples of students’ insights and approaches to managing uncertainty. For example, students reported that they held a better understanding of the different strengths and perspectives a team can bring to meet a challenging situation, again a finding that translates well into the clinical setting. However, not all students enjoyed, or perceived that they had learned from, the DEER. For example, students that had encountered technology problems during game-play were less positive about the experience overall. This highlights that issues such as internet access and digital skills represent an important challenge for DEERs in comparison to physical escape rooms. Furthermore, quantitative data analysis found no evidence that the DEER had had an impact on the students’ uncertainty tolerance. It may be that a once-off intervention or a short interval between measurement was insufficient to detect a change in students’ responses. The small cohort of this pilot study makes it difficult to draw firm conclusions.

We also set out to explore whether or not the CoI framework could facilitate our understanding of DEER learning environments, and, if so, what indicators of social presence, teaching presence and cognitive presence exist. Our findings strongly suggest that CoI has a natural resonance with DEER learning environments, and that the framework can shed light on how learning takes place in such novel online settings. We also found evidence of cognitive, teaching and social presences that we will discuss in relation to the existing literature below.

Social presence, which relates to open communication, emotional expression, and group cohesion [51], was widely evident within the participants’ interactions. The DEER seemed to encourage rapid rapport and trust building, and despite some initial hesitation about playing the game with unfamiliar individuals, they quickly settled into teamwork. This was particularly apparent in the breakout rooms where, in the absence of the instructor, participants engaged in supportive, informal and humour-filled verbal communication. This finding supports previous CoI research [59, p.6], which suggests that ‘synchronous communications can be especially useful in quickly establishing, building and modeling social presence.’ There were also many, varied expressions of affective experiences during game play. Aside from uncertainty, students reported feeling enjoyment, humour, curiosity and pride, as well as anxiety and frustration. These findings support evidence that DEERs can offer learners opportunities ‘to deal with and overcome intense negative emotions, in particular fear or disgust, to move forward’ [60, p.16], which may be particularly useful in preparing medical students for ‘emotion-laden’ clinical experiences [61, p.198].

Teaching presence was also evident within the escape room environment, with different aspects apparent at different stages of the game. For example, teaching presence centred on the instructor during preparation for the game and within the pre- and de-brief sections. Teaching presence centred on peer interaction was most apparent in the small-group breakout rooms. This finding underlines a view within CoI research that ‘the term for this component of the CoI is ‘teaching’ and not ‘teacher’ presence. This provides room for, and encourages, students to take a positive and visible role in the learning of their peers’ [59, p.7], The extension of teaching presence to embrace students as teachers has been proposed as a ‘vital question’ which should be addressed as the CoI model matures [62, p.27] Our findings suggest that DEERs can provide a valuable learning environment for peer learning which may help student to understand the salience of ‘building relationships with staff, peers or near-peers’ in clinical settings [3, p.566]. ‘Students as teachers’ also hints at a potential for DEER activities be scaled up, offering an effective vehicle for active learning in online, large group classrooms. To do so, it may be helpful for educational game designers to consider including opportunities for students to take on instructional roles when planning game-play strategies.

Indicators and examples of cognitive presence were also apparent within the DEER, although fewer in number. This is not surprising considering that cognitive presence, which represents a critical-thinking process that switches ‘between the public shared world and the private reflective world’ [25, p.21], can be hard to observe. Here, it appeared that the emotional arousal elicited by the puzzles drew most students into a cycle of cognitive activity. At times this activity seemed aligned with the deep processes involved in cognitive presence but, at others, it seemed more superficial. It is worth highlighting that lively interaction may be present in a learning environment, but if it does not support participants to integrate ideas into meaningful constructs, it does not represent the existence of cognitive presence [63]. This finding may be due to the design of this specific DEER, i.e. here the aim was to provoke experiences of, and reflections on, uncertainty, rather than present content material that provoked deeper cognitive processing. Nonetheless, our results suggest that strong alignment of game-play and puzzle content with learning outcomes is advisable.

Other elements of the game design also seemed to impact on cognitive presence. For example, the in-game reflective activities encouraged some students to engage in shared reflection, whilst triggering annoyance and frustration for others. Furthermore, the game’s time strategy seemed to impact on the students’ approaches to puzzles in different ways. Some groups found the time limit exciting, whilst others experienced it as pressure, causing them to skip over the activities. This tension between achieving game goals and engaging in deep, reflective learning in a time-constrained game environment has been highlighted in the literature [64,65]. Thus, whilst our findings suggest that DEERs offer advantages in keeping learners ‘on-task’ in the online setting, care must be taken to ensure that puzzles and game-play align with intended learner behaviour and meaningful learning outcomes, which award students with a ‘sense of purpose’. For example, a limited-time strategy that encourages students to ‘race to the finish’ might be useful for exploring a clinical scenario where quick action is required (e.g., managing sepsis); however, the sense of urgency this evokes may divert students away from the sustained communication required for cognitive presence [65].

### Limitations and future research

Our study population represented a small convenience sample of medical students. It is likely that our participants were inherently interested in educational escape games, and a larger cohort of participants may have led to different findings. A larger sample size would also be helpful in identifying any statistically significant changes between the pre- and post-intervention questionnaire responses. To deepen our understanding of how the CoI framework can be used in the design and implementation of DEERs, we recommend that further research is carried out in different contexts, with different DEER formats and diverse populations of students. A future prototype of this DEER will be incorporated into the medical programme at RCSI University of Medicine and Health Sciences, providing an opportunity to test our proposed design principles, and to evaluate the scalability of the intervention in a large group classroom. More broadly, this study highlights the opportunities provided by DBR in supporting the development of educational resources, alongside gaining insight as to how these operate within specific learning environments. DBR may be of specific interest to health professions’ educators who wish to investigate the application of innovations such as virtual reality, augmented reality and artificial intelligence within real-world settings.

## Conclusion

Overall, our study suggests that DEERs offer a suitable learning environment for medical students to engage with complex, team-based and emotion-provoking challenges, such as those experienced in the transition from pre-clinical to clinical training. Our findings also support the value of CoI as a lens through which the DEER learning environment can be explored. The framework has highlighted important considerations in the advancement of this specific prototype, as well as offering more general guidance with respect to the cultivation of engaging, collaborative DEER learning environments. We concur with McKerlich and Anderson’s [48, p.48] assertion that CoI offers a valuable way to ‘describe and assess educational experiences and contexts’. As research around game-based learning and simulation games expands, these approaches are likely to gain ground on more traditional pedagogical methods in health professions’ education.

## Appendices

**Appendix A:** Focus group question guide

https://docs.google.com/document/d/1dNrzDRuPP9006W2cB4duHX9T4vqjt5WS/edit?usp=sharing&ouid=113531471089624837741&rtpof=true&sd=true

**Appendix B:** Pre-intervention questionnaire

https://docs.google.com/document/d/1-cDDSVXkFX240HyWn515exJYzpWjMFYH/edit?usp=sharing&ouid=113531471089624837741&rtpof=true&sd=true

**Appendix C:** Post-intervention questionnaire

https://docs.google.com/document/d/12WuUWhNUJ7TtlNLtBRCsrcaThdduXjJV/edit?usp=sharing&ouid=113531471089624837741&rtpof=true&sd=true

**Appendix D:** Indicators and examples of cognitive, teaching and social presence derived from focus group discussions and game-play sessions

https://docs.google.com/document/d/16Q6y7nQwPzxJGYD8lykhr9ftfVYmoWeV/edit?usp=sharing&ouid=113531471089624837741&rtpof=true&sd=true
